# Book Reviews

**Published:** 1918-09

**Authors:** 


### BOOK REVIEWS

Books mentioned may be inspected at and ordered through this office. So far as possible, books received in any month will be reviewed in the issue of the second month following. Pamphlets, quarterly and similar periodicals, reports, transactions, etc., will, as a rule, merely be mentioned.

**Diseases of the Male Urethra Including Impotence and Sterility.** By Irvin S. Koll, B. S., M. D., F. A. C. S. Professor of Genito-urinary Diseases, Post-Graduate Medical School and Hospital; Associate Genito-urinary Surgeon, Michael Reese Hospital, Chicago. Octavo of 151 pages, with 123 illustrations, several in colors. Philadelphia and London: W. B. Saunders Company, 1918. Cloth, $3.00 net.

Fully one-half of this monograph is devoted to the consideration of gonorrhea, one of the most discussed of genito-urinary diseases, but which at the same time from a therapeutic standpoint has progressed the least. The author well expressed his attitude in the preface by saying,—"I realize full well my views are reacting and may not be shared by some of my colleagues." In spite of this the author has prepared a commendable work which will be of great value to the student and the practitioner because of clear, simple and concise treatment. The author carefully covers the anatomy, the symptomatology, the complications on impotence and on sterility with their causation and treatment.

The reviewer realizes that unfortunately the author has not added anything which might prove useful in the treatment of gonorrhea. His treatise is well done, enhanced by many carefully prepared illustrations descriptive of the condition that gives definite pictures to the man who is in a hurry and needs to know quickly what to do.

---

**The Seriousness of Venereal Disease.** By Sprague Carleton, M. D., F. A. C. S. Illustrated, New York, Paul Hoeber, 1918. Pp. 67. Price, $.50.

The attempt of the author at this time of social disturbance due to war conditions, is laudable. Very little is said; the volume is simply an exhibit of pictures of syphilis, chancroid, bubo, etc., with a very short descriptive report of the case and the medical and moral lesson to be drawn therefrom; just enough to hold the attention of the soldier and give him some idea of the disease. The photographs represent the diseases as they are without exaggeration; if anything they underestimate the seriousness of the disease as many of the pictures lack illuminating detail. The reviewer is of the opinion that in a book ostensibly written for the soldier it would have been better to have made the announcement of its specific purpose. The author's effort is worth the while and while not remarkable, it will serve a useful purpose in enlightening those ignorant of the havoc of venereal diseases.

---

**Clinical Diagnosis.** A Manual of Laboratory Methods. By James Campbell Todd, M. D., Professor of Pathology, University of Colorado. Fourth edition, revised and reset. 12mo of 687 pages with 232 text-illustrations and 12 colored plates. Philadelphia and London: W. B. Saunders Company, 1918. Cloth, 14s. net.

That the fourth edition of Todd's popular book is received so soon after the publication of the third edition is the very strongest commendation of its usefulness to the profession. The book has been somewhat extended in scope and matter and the size in consequence has been increased. His position as a teacher, well fits the author to present his subject in a rational and practical way with enough detail to make the tests easily carried out by the student and general practitioner for whom the work primarily was prepared.

The laboratory methods especially considered are those that have a clinical value. As it is not designed for the trained laboratory worker, the methods offered are simple and practical, emphasis being placed as far as possible on those requiring little complicated apparatus. The quantity and quality of the new material added is of special interest and brings up to the minute laboratory diagnostic technique useful to and available for the general practitioner. The changes and additions are widely scattered throughout the book. They include the use of colorimeters; the spectroscope; methods of matching blood for transfusion; the new Bass and Johns concentration methods for malarial parasites; the fractional method of gastric analysis; vital blood staining; the Wilber and Addis method for urobilin in the diagnosis of pernicious anemia; and, the estimation of amylase in urine and feces in pancreatic diseases.

The illustrations have been materially increased; the black and white pictures have received special consideration with the elimination of the poor in former editions and the addition of at least 90 new ones. These changes and additions give accurate pictures of microscopic structures thus making interpretation easy and giving information that can easily be remembered.

The book is fully entitled to the flattering reception accorded previous editions.

